# A comparison of perioperative outcome between robot-assisted and laparoscopic radical prostatectomy: experience of a single institution

**DOI:** 10.1590/S1677-5538.IBJU.2018.0367

**Published:** 2019-09-02

**Authors:** Feng Qi, Shangqian Wang, Haoxiang Xu, Yiren Gao, Gong Cheng, Lixin Hua

**Affiliations:** 1 Department of Urology, The First Affiliated Hospital of Nanjing Medical University, Nanjing, China

**Keywords:** Robotics, Laparoscopy, Prostatic Neoplasms

## Abstract

**Purpose:**

To compare perioperative and pathological results in different approaches of robotic or laparoscopic radical prostatectomy.

**Materials and Methods:**

We retrospectively reviewed 206 patients diagnosed with prostate cancer (PC) from June 2016 to October 2017 in the First Affiliated Hospital of Nanjing Medical University. A total of 132 cases underwent robot-assisted laparoscopic radical prostatectomy (RLRP) including 54 patients on transperitoneal robot-assisted laparoscopic radical prostatectomy (Tp-RLRP) and 78 on extraperitoneal robot-assisted laparoscopic radical prostatectomy (Ep-RLRP). Meanwhile, 74 patients performed with extraperitoneal laparoscopic radical prostatectomy (Ep-LPR) were also included. Perioperative and pathological data were compared among these groups.

**Results:**

All operations were completed without conversion. There was no significant difference in basic and pathological characteristics of patients between each two groups. *In Tp-RLRP vs. Ep-RLRP:* Significant differences were found in the comparison in total operation time [235.98 ± 59.16 vs. 180.45 ± 50.27 min, P = 0.00], estimated blood loss (EBL) [399.07 ± 519.57 vs. 254.49 ± 308.05 mL, P = 0.0473], postoperative pelvic drainage time [5.37 ± 2.33 vs. 4.24 ± 3.08 d, P = 0.0237] and postoperative length of stay [8.15 ± 3.30 vs. 6.49 ± 3.49 d, P = 0.0068] while no significant differences were detected in other variables. *In Ep-RLRP vs. Ep-LPR:* Longer total operation time was observed in Ep-RLRP when compared to Ep-LPR [180.45 ± 50.27 vs. 143.80 ± 33.13 min, P = 0.000]. No significant differences were observed in other variables.

**Conclusion:**

In RLRP, Ep-RLRP was proved a safe and effective approach based on the perioperative results compared to Tp-RLRP. Ep-RLRP and Ep-LPR provides equivalent perioperative and pathological outcomes.

## INTRODUCTION

Prostate cancer (PC) is the second most common malignant tumor in men and an important cause of cancer-related morbidity and mortality worldwide. In 2017, the estimated new PC cases and deaths were 161.360 and 26.730 in the United States, respectively (1). Generally, surgery is the standard of care for the treatment of localized disease to achieve an extended life expectancy.

Before minimally invasive surgery was widely used, open radical prostatectomy (ORP) had been a good alternative for the treatment of PC. However, high incidence of iatrogenic diseases caused by open surgery have led people to look for minimally invasive ways to improve perioperative and postoperative conditions.

Laparoscopic radical prostatectomy (LRP) technique was first systematically reported in 1997 by Schuessler (2) and relevant studies showed that this technique provides better perioperative and postoperative outcomes compared to ORP (3-5). In 2001, the first robot-assisted laparoscopic prostatectomy (RLRP) was reported by Binder and Kramer (6); since then the rapid development of RLRP has made it an important surgical alternative for prostatectomy in many countries. In the United States, 60% of prostatectomies were performed with RLRP in 2007 (7). Presently, there are two approaches for RLRP: transperitoneal robot-assisted laparoscopic radical prostatectomy (Tp-RLRP) and extraperitoneal robot-assisted laparoscopic radical prostatectomy (Ep-RLRP).

Although there are some previous comparisons between LRP and RLRP (8-10), the surgical approach was always unclear, like a mixture of transperitoneal and extraperitoneal, data were limited on the comparisons of extraperitoneal LRP (Ep-LRP) vs. Ep-RLRP and Tp-RLRP vs. Ep-RLRP. Therefore, a single-center retrospective analysis was performed in patients diagnosed with localized PC who underwent RLRP or LRP from June 2016 to October 2017.

## MATERIALS AND METHODS

### Patient Selection

From June 2016 to October 2017, patients diagnosed with organ-confined PC who underwent LPR or RLRP in our institution were included in our study. Patients were excluded from this research if they had had any other malignant tumors and serious diseases. All patients were newly diagnosed and had not received other treatments for PC before, such as brachytherapy, external radiotherapy, chemotherapy, etc. A total of 206 patients were selected into 3 groups (Ep-LRP, Tp-RLRP and Ep-RLRP). Detailed basic characteristics of patients in each group are summarized in Table-1.

**Table 1:** Basic characteristics of patients in Ep-LRP, Ep-RLRP and Tp-RLRP.

	Tp-RLRP	Ep-RLRP	Ep-LRP
N	54	78	74
Age, median (range), year	72.5 (56-80)	66 (48-79)	69 (68-81)
BMI, median (range), kg/m^2^	23.9 (17.8-30.8)	24.35 (14.5-30.1)	24.05 (17.5-29.8)
**Biopsy GS, n (%)**			
≤6	2 (3.7%)	11 (14.1%)	16 (21.6%)
7	26 (48.1%)	41 (52.6%)	40 (54.1%)
>7	26 (48.1%)	26 (33.3%)	18 (24.3%)
**Perioperative PSA, n (%)**			
≤10	17 (31.5%)	22 (28.2%)	22 (29.7%)
10-20	16 (29.6%)	29 (37.2%)	23 (31.1%)
≥20	21 (38.9%)	27 (34.6%)	29 (39.2%)
**Clinical T stage, n (%)**			
T1-T2	38 (70.4%)	63 (80.8%)	60 (81.1%)
T3-T4	16 (29.6%)	15 (19.2%)	14 (18.9%)
A prior history of abdominal surgery, n (%)	13 (24.1%)	20 (25.6%)	20 (27.0%)
A prior history of TURP, n (%)	3 (5.6%)	5 (6.4%)	6 (8.1%)

**BMI =** Body Mass Index; **PSA =** prostate specific antigen; **Tp-RLRP =** transperitoneal robot-assisted laparoscopic radical prostatectomy; **Ep-RLRP =** extraperitoneal robot-assisted laparoscopic radical prostatectomy; **Ep-LRP =** extraperitoneal laparoscopic radical prostatectomy; **TURP =** transurethral resection of prostate; **GS =** Gleason score

### Surgical Technique

Ep-LRP: Patients were placed in the supine position; five ports were used in the operation. The position for each trocar and general surgical procedures were described previously (11).

Tp-RLRP: Patients were placed in the half lithotomy position with their legs outreached at 30° higher than head level. The position for each trocar and general surgical procedures were described previously (12).

Ep-RLRP group: Unlike the transperitoneal approach, five ports were used lower in the pelvis. The position for each trocar and general surgical procedures were described previously (13).

Laparoscopic technique has been carried out for nearly ten years and robot-assisted operation was developed successfully in our institution based on the mature laparoscopic technique. There was no specific indication for one technique to another, and main influence factors for choosing surgery techniques were patient’s will and figure. Whether transperitoneal or extraperitoneal surgical approach (with or without robotic assistance) were all common surgery styles without learning curve effect.

Patients diagnosed with PC by biopsy underwent surgery at 6-8 weeks after biopsy in order to reduce the difficulty of surgery and postoperative complications. Additionally, patients who underwent transurethral resection of prostate (TURP) should wait 12 weeks for further surgery. All operations were performed by the same surgeon who has worked for 30 years and has been involved in PC surgery. Since 2009, he has completed more than 1.000 cases of LRP (more than 100 cases of RLRP). Additionally, postoperative management for each patient was the same, regardless in LRP or RLRP. In all surgeries, pelvic lymph node dissection (PLND) was performed in patients with a serum prostate specific antigen (PSA) greater than 10 ng / mL, or biopsy Gleason score (GS) more than 7. In general, the range of PLND includes the nodes covered on external iliac arteries and veins, the nodes within the obturator and the nodes overlying internal and external side of internal iliac arteries (14). Moreover, nerve-sparing procedure was performed according to preoperative evaluation, such as age, tumor clinical grade, magnetic resonance Imaging (MRI) evaluation, International Index of Erectile Function (IIEF) score.

### Data extraction

All data analyzed in this study were based on the documentations of our PC database including age, body mass index (BMI), preoperative PSA, biopsy GS, prior history of abdominal surgery, estimated blood loss (EBL), total operation time, postoperative pelvic drainage time, the indwelling catheter time, postoperative length of stay, extra-prostatic extension (EPE), lymph node invasion (LNI) and cases of seminal vesicle and vas deferens involved. The pathological results including postoperative GS and positive surgical margin (PSM) were also documented.

### Statistical analysis

Stata software (version 12.0; StataCorp LP, College Station, TX) was used for the statistical analysis. Pearson’s chi-square test was used for the comparison of nominal data while the numeric parameters were compared utilizing Student’s t-test. For all analyses, two-sided P value < 0.05 was considered as statistical significant.

## RESULTS

### Preoperative data

All operations were completed successfully without conversion. No significant differences in preoperative data were detected between every two groups except the comparison in age between Tp-RLRP and Ep-RLRP which may due to the relatively small sample size (Table-2).

**Table 2:** Comparisons in preoperative data between each two groups (Ep-LRP vs Ep-RLRP and Ep-RLRP vs Tp-RLRP).

	Tp-RLRP	Ep-RLRP	P value	Ep-RLRP	Ep-LRP	P value
Age (mean±SD), year	70.5±6.23	66.77±7.12	0.0024	66.77±7.12	68.96±7.34	0.0638
BMI (mean±SD), kg/m2	23.98±2.56	24.19±2.83	0.6599	24.19±2.83	24.06±2.81	0.7761
Biopsy GS (mean±SD)	7.5±0.75	7.24±0.76	0.0569	7.24±0.76	7.04±0.71	0.0912
Perioperative PSA (mean±SD), ng/mL	24.51±24.55	24.17±25.72	0.9396	24.17±25.72	26.62±29.74	0.5879
Clinical T stage, n (%)						
T1-T2	38 (70.4%)	63 (80.8%)	0.416	63 (80.8%)	60 (81.1%)	0.951
T3a	3 (5.6%)	5 (6.4%)		5 (6.4%)	4 (5.4%)	
T3b	11 (20.4%)	8 (10.3%)		8 (10.3%)	7 (9.5%)	
T4	2 (3.7%)	2 (2.6%)		2 (2.6%)	3 (4.1%)	
A prior history of abdominal surgery, n (%)	13 (24.1%)	20 (25.6%)	0.838	20 (25.6%)	20 (27.0%)	0.846
A prior history of TURP, n (%)	3 (5.6%)	5 (6.4%)	0.839	5 (6.4%)	6 (8.1%)	0.686

**PSA =** prostate specific antigen; **GS =** Gleason score; **Tp-RLRP =** transperitoneal robot-assisted laparoscopic radical prostatectomy; **Ep-RLRP =** extraperitoneal robot-assisted laparoscopic radical prostatectomy; **Ep-LRP =** extraperitoneal laparoscopic radical prostatectomy; **TURP =** transurethral resection of prostate; **SD=** standard deviation

### Perioperative outcome and pathological results

Detailed information of comparison between each two groups is shown in Table-3. The whole LNI, EPE rate and PSM was 7.3%, 36.4% and 42.2%, respectively.

**Table 3:** Comparisons in perioperative and pathologically data between each two groups (Ep-LRP vs Ep-RLRP and Ep-RLRP vs Tp-RLRP).

	Tp-RLRP	Ep-RLRP	P value	Ep-RLRP	Ep-LRP	P value
operation time (mean±SD), min	235.98±59.16	180.45±50.27	0.000	180.45±50.27	143.80±33.13	0.000
EBL (mean±SD), mL	399.07±519.57	254.49±308.05	0.0473	254.49±308.05	316.89±200.73	0.1433
postoperative length of stay (mean±SD), day	8.15±3.30	6.49±3.49	0.0068	6.49±3.49	7.09±5.68	0.4255
the indwelling catheter time, (mean±SD), day	11.52±1.47	11.73±2.88	0.6164	11.73±2.88	12.85±5.04	0.0924
cases of seminal vesicle involved, n (%)	11(20.4%)	8(10.3%)	0.104	8(10.3%)	7(9.5%)	0.869
postoperative pelvic drainage duration time (mean±SD), day	5.37±2.33	4.24±3.08	0.0237	4.24±3.08	4.77±5.69	0.4705
PSM, n (%)	19 (35.2%)	34(43.6%)	0.333	34(43.6%)	34(45.9%)	0.770
postoperative GS	7.35±0.87	7.35±0.98	0.9726	7.35±0.98	7.45±0.83	0.4998
EPE rate, n (%)	13 (24.1%)	30 (38.5%)	0.083	30 (38.5%)	32 (41.2%)	0.549
PLND, n (%)	16 (29.6%)	32 (41.0%)	0.178	32 (41.0%)	33 (44.6%)	0.657
LNI rate, n (%)	3 (5.6%)	5 (6.4%)	0.840	5 (6.4%)	7 (9.5%)	0.486

**EBL =** estimated blood loss; **PSM =** positive surgical margin; **GS =** Gleason score; **Tp-RLRP =** transperitoneal robot-assisted laparoscopic radical prostatectomy; **Ep-RLRP =** extraperitoneal robot-assisted laparoscopic radical prostatectomy; **Ep-LRP =** extraperitoneal laparoscopic radical prostatectomy; **EPE =** extra-prostatic extension; **PLND =** pelvic lymph node dissection; **LNI =** lymph node invasion; **SD =** standard deviation

### Ep-LRP vs. Ep-RLRP

Longer total operation time [180.45 ± 50.27 vs. 143.80 ± 33.13 min, P = 0.000] was found in Ep-RLRP when compared to Ep-LRP. Additionally, no statistical difference was found in other variables.

### Tp-RLRP vs. Ep-RLRP

Significant differences were detected in the comparison of EBL [399.07 ± 519.57 vs. 254.49 ± 308.05 mL, P = 0.0473], total operation time [235.98 ± 59.16 vs. 180.45 ± 50.27 min, P = 0.00], postoperative pelvic drainage time [5.37 ± 2.33 vs. 4.24 ± 3.08 d, P = 0.0237] and postoperative length of stay [8.15 ± 3.30 vs. 6.49 ± 3.49 d, P = 0.0068] between Tp-RLRP and Ep-RLRP while difference in the comparisons of other variables showed no statistical significance.

## DISCUSSION

PC is a male malignant tumor with high incidence (1). Definitive treatment for localized PC includes surgery, radiation therapy, endocrine therapy, active surveillance and watchful waiting. However, radical prostatectomy has been a recognized method for relatively young patients with a life expectancy over 10 years (15). Because of the decreased EBL, shorter length of stay and less postoperative pain that the minimally invasive techniques provide in radical prostatectomy compared to open surgery (16), radical prostatectomy has been always performed in the form of LRP or RLRP in recent years.

There are some typical features favored in RLRP such as 3D viewing, improved ergonomics, elimination of hand tremor and refined dexterity (17, 18), which had made RLRP a good alternative for radical prostatectomy. However, high cost, lack of training and reduced budgets of RLRP became the biggest obstacle to its development (19). Similarly, in partial nephrectomy, many studies had shown that robotic-assisted laparoscopic partial nephrectomy is only a viable approach rather than an absolutely better one due to equivalent postoperative outcomes (20) and greater economic burden and when compared to laparoscopic partial nephrectomy. Also, some researchers considered RLRP as a product of market profit due to the lack of advanced evidence of surgical advantage (21, 22).

Traditionally, radical prostatectomy can be performed via transperitoneal and extraperitoneal approach. Either a transperitoneal or an extraperitoneal approach have been proved to be safe and eﬀective, and each approach has its advantages and short comes. The main advantages for transperitoneal approach are summarized as following: (1) easier for trocars placement; (2) the larger operation space for procedure, like the placement of a specimen bag and a broader surgical field. However, a steeper Trendelenburg position may lead to upper airway and facial swelling, which may result in worse postoperative recovery (23). The extraperitoneal approach has several advantages: (1) less steep Trendelenburg position can lead to the lower incidence rate of intestinal and peritoneal diseases; (2) Isolation of the operating fields from abdominal cavity can avoid the occurrence of reflex ileus and urinary ascites followed by bleeding to the abdominal cavity (24, 25). However, the risk of injury to the rectum during seminal vesicles dissection increases.

In our study, operation time, postoperative pelvic drainage time, postoperative length of stay and less EBL was favored in Ep-RLRP when compared to Tp-RLRP. Comparison between Ep-LRP and Ep-RLRP showed no statistical difference except the longer total operation time in Ep-RLRP.

The operation time, defined as a period of time from the incision of the skin to the end of the skin suture, was different in various surgical approaches. Significant difference (P < 0.0001) between Ep-RLRP and Ep-LRP could have been caused by the extra time for disposition of robot arms. Longer operation time in Tp-RLRP when compared to Ep-RLRP may have occurred due to faster placement of trocars (P < 0.0001).

In terms of EBL, patients with Tp-RLRP had more blood loss than those of Ep-RLRP (399.07 vs. 254.49 mL, P = 0.0473). However, no significant difference was observed in the comparison between Ep-RLRP and Ep-LRP. Therefore, a preliminary conclusion can be drawn that more EBL is tightly associated with the transperitoneal route, similar results can be found in some previous studies which compare EBL between Ep-LRP and Tp-LRP (25-27). One possibility is that a self-made gas bag can make enough pressure on the surrounding tissue to lower the bleeding in extraperitoneal route (28).

In the comparison between Tp-RLRP and Ep-RLRP, we can conclude that the postoperative length of stay and pelvic drainage duration time was significant longer in Tp-RLRP. This might be explained by the disadvantages of Tp-RLRP mentioned above. However, the difference between Ep-LRP and Ep-RLRP showed no statistical significance which indicated that the robot-assisted technique did not have especially obvious effect on postoperative recovery.

Generally, postoperative pathological results were tightly to PSM and postoperative GS. PSM is an independent predictor of tumor progression which can probably be prevented by appropriate patient selection and meticulous surgical technique (29). In our study, no significant differences were observed in PSM and postoperative GS in each two groups. As the results of Hakimi et al. (30) and Eden et al. (11) research, which compared PSM in (LRP vs. RLRP) and (ELRP vs. TLRP), showed no statistical significance in the comparison of PSM. However, the relatively small sample size and the lack of long-term follow-up data of biochemical recurrence limited the evaluation of postoperative conditions; larger sample size and longer follow-up are needed. The relatively high PSM rate (42.23%) in this series should not be ignored. We reviewed the biopsy GS and pre-operative PSA of all patients included and found that most patients were in or above intermediate risk, besides, the extra-prostatic extension rate suggested the similar results in postoperative pathology. Certainly, the small sample size may also have played a role.

In Table-3, we can found that postoperative duration of catheter was relatively long in our instruction and the pelvic drainage is today rarely routinely placed in many centers. Firstly, we attributed the longer duration of catheter to the different concepts we told to patients, what’s mean that we will try to prolong the duration of catheter as slightly as possible (while ensuring no infection) to ensure a better anastomosis between the urethra and the bladder, and to reduce the incidence of anastomotic leakage and urinary failure after extubation. Secondly, there have been many reports on postoperative pelvic drainage and they mentioned that incidence of adverse events in the no drain group was not inferior to the group who received a pelvic drainage (31). However, placement of drainage tubes is a generally accepted concept in China. Additionally, Patel et al. (32) believes that the contents of the drainage tube can provide additional information after surgery, potential bleeding and leakage of urine or serious complications can be detected earlier through the observation of the color and volume of the drainage or the inspection of the drainage if necessary. Moreover, the drainage tube can reduce the formation of postoperative hematoma, and patients with hematoma have long been confirmed to have a large proportion of bladder neck contracture and permanent urinary incontinence (33).

This was a single-instruction, retrospective study, and no strict selection criteria were applied when choosing the surgery technique (almost to be a randomized clinical trial). The surgeon has already been an experienced operator, and we thought the bias of experience accumulation can be minimized. The limitation for this study could be overcome by expanding the number of cases in each group with a longer follow-up period in future studies.

## CONCLUSIONS

In RLRP, Ep-RLRP was proved to be a safe and effective approach because of the shorter operation time, postoperative pelvic drainage time, postoperative length of stay and less EBL when compared to Tp-RLRP. Ep-RLRP and Ep-LPR provides equivalent perioperative and pathological outcomes.
